# “We have no safety at all”: insecurity and continuous traumatic stress among Palestinian adolescents in East Jerusalem

**DOI:** 10.3389/fpsyg.2025.1665579

**Published:** 2025-09-26

**Authors:** Heba F. Zedan

**Affiliations:** Children and Youth Rights Program, Minerva Center for Human Rights, Faculty of Law, Hebrew University of Jerusalem, Jerusalem, Israel

**Keywords:** continuous traumatic stress, Palestinian youth, participatory research, children’s rights, occupation, East Jerusalem, political violence

## Abstract

This research was completed in collaboration with the Children and Youth Advisory Group: Mays, Nada, Amro, Sajida, Kinan, Rama, and Qamar.

**Introduction:**

Children and youth in conflict zones are often exposed to persistent sociopolitical stress that undermines their rights to protection and development. This study explores how Palestinian adolescents in occupied East Jerusalem experience and respond to continuous traumatic stress amid intensified political violence during the ongoing Gaza war.

**Methods:**

Using a participatory research approach, an advisory group of youth co-researchers contributed to study design, ethics, data collection, and analysis. In-depth interviews were conducted with 24 Palestinian youth aged 12–19, alongside eight adult parents and professionals.

**Results:**

Reflexive thematic analysis revealed six interconnected themes: (1) Perpetual threat in everyday spaces; (2) Adaptive hypervigilance; (3) Collective and intergenerational transmission of stress; (4) Emotional suppression and helplessness; (5) Normalization of abnormality; and (6) Distrust in protective systems. The findings demonstrate how structural violence is internalized, embodied, and transmitted across generations, creating a persistent emotional climate of fear and insecurity.

**Conclusion:**

This study calls for trauma frameworks that move beyond episodic models to address cumulative, politically rooted stressors that violate children’s rights under international law. Policies must prioritize rights-based interventions, including accessible psychosocial support and protective legal frameworks that counter systemic oppression, surveillance, and discrimination. Integrating children’s lived experiences and agency into service design and delivery is highly recommended.

## Introduction

Children and adolescents^1^ growing up in militarized and politically contested environments such as occupied East Jerusalem experience chronic sociopolitical stressors that profoundly shape their developmental trajectories and mental health. Armed conflict not only threatens their physical safety but also erodes their well-being by undermining foundational needs for security, stability, and dignity (Atkinson et al., 2014; Wentz et al., 2023). These disruptions are especially acute for children and youth, whose developmental sensitivity, evolving identities, and limited political agency heighten vulnerability to long-term consequences of violence, displacement, and exclusion (Cummings et al., 2017).

Palestinian children in East Jerusalem face persistent legal and social precarity that exposes them to racialized policing, economic disenfranchisement, and threats to both individual safety and collective identity (Veronese et al., 2022). Although residing within Jerusalem’s municipal boundaries, and considered permanent residents of sovereign Israel, their lived experiences reflect deep inequities that remain largely unacknowledged in dominant global trauma and child development discourses (Kovner, 2020). These children experience a unique and enduring set of structural threats shaped by the ongoing Israeli occupation. These include home demolitions, systematic surveillance, mobility restrictions, unequal access to education and healthcare, and frequent armed confrontations (Aly et al., 2025). Such conditions are not episodic or incidental but are embedded within a broader settler-colonial framework that institutionalizes ethnic hierarchies. The 2018 Nation-State Law, for example, constitutionally enshrines Jewish self-determination, reinforcing the marginalization of Palestinians with formal citizenship as second-class residents (Tanous et al., 2022). Consequently, children and adolescents in East Jerusalem face continuous, identity-targeted insecurity, not isolated traumatic “events” that shapes how they perceive danger, regulate emotion, and make developmental choices. Yet most research and practice still rely on models designed for discrete events and individual treatment, which miss how threat is constant, cumulative, and structurally produced. A youth-centered, rights-based, and Continuous Traumatic Stress (CTS) theory-informed study is needed to document how youth themselves describe risk, safety, and coping under these conditions, especially amid the escalation of the ongoing war on Gaza. This study explores the perceptions of Palestinian adolescents in East Jerusalem and responses to chronic sociopolitical stress, drawing on a participatory qualitative design and thematic analysis of semi-structured interviews. The study’s practical, theoretical, and methodological contributions are discussed.

## Literature review

A growing body of research documents how military occupation and structural violence affect children’s mental health and social development (e.g., Qouta et al., 2008; Sagi-Schwartz, 2008; Veronese et al., 2022). Daily exposure to surveillance, arrest, intimidation, and movement restrictions—often normalized by “necessity”—leads to complex psychological adjustments that blur the line between adaptation and survival (Veronese et al., 2022). Israeli authorities treat Palestinian youth as security risks rather than rights-bearing individuals, exacerbating their vulnerability and obstructing access to age-appropriate protections and care (Kovner, 2020). Importantly, these conditions represent not isolated crises but continuous, cumulative exposures that affect all aspects of everyday life.

The ongoing Gaza war following has significantly exacerbated state and settler aggression in East Jerusalem and the West Bank (Aly et al., 2025). Human rights organizations note a dramatic rise in settler attacks and military raids during this period—marked by airstrikes, live-fire raids, arrests, and increased curfews and checkpoints— describing it as the “Gazafication” of the West Bank. This ongoing intensification of the conflict further destabilizes everyday life for Palestinian children and families (B’Tselem, 2025).

These lived realities reflect ongoing violations of the United Nations’ Convention on the Rights of the Child (UNCRC), including the rights to protection, education, movement, expression, and freedom from degrading treatment (Articles 13, 15, 19, 28, 37, respectively; United Nations, 1989). While such violations are well-documented, less is known about how children themselves experience and respond to these threats. In protracted conflict zones such as East Jerusalem, conceptualizing children’s rights requires a nuanced understanding that addresses both their immediate need for protection and their long-term well-being. Peleg (2013) advances this perspective through a “hybrid childhood” model that aligns with the CRC’s principle of the right to development, emphasizing that development is inseparable from safety, participation, and the capacity to build resilience. Drawing on the capability approach, he argues that even in contexts of chronic threat, children must be recognized not only as victims, but as agents capable of meaningful participation and of shaping their own developmental trajectories – an approach adopted in the present study.

### Conceptual framework: continuous traumatic stress and identity-based trauma

This study is anchored in the theory of continuous traumatic stress (CTS; Eagle and Kaminer, 2013), which conceptualizes traumatic stress in settings where threat is ongoing, inescapable, and structurally maintained—conditions under which safety-oriented hypervigilance (often labeled “hypervigilance” in PTSD models) emerges as an adaptive and persistent response to the absence of safety. CTS theory offers a powerful lens for interpreting Palestinian children’s lived experiences, as it centers the subjective perception of ongoing, inescapable threat rather than isolated traumatic events. In this study, we first conducted inductive, participant-led analysis; following initial coding, we adopted CTS as the primary interpretive lens. CTS therefore guides (a) our second-cycle analysis and naming of patterns and (b) our interpretation and practice/policy implications, while data-collection procedures were not designed *a priori* around CTS. By validating adaptive responses such as hypervigilance and emotional suppression, it situates children’s insecurity within the structural realities of occupation and violence, rather than framing it as pathological or irrational. CTS theory provides a context-sensitive framework that challenges conventional trauma models—such as PTSD—which assume a discrete traumatic event followed by a period of recovery. Developed in post-apartheid South Africa and since applied to militarized and structurally violent settings, CTS focuses on the real-time psychological impact of persistent, inescapable danger (Hecker et al., 2017; Kira et al., 2013; Nuttman-Shwartz and Shoval-Zuckerman, 2016).

A hallmark of CTS is its emphasis on cyclical and adaptive trauma responses in persistently threatening environments. It highlights hypervigilance (conceptualized here as safety-oriented vigilance or adaptive hypervigilance), emotional suppression, avoidance, and anticipatory cognitive responses not as pathological symptoms, but as functional survival strategies in chronically unsafe conditions (Hecker et al., 2017; Nuttman-Shwartz and Shoval-Zuckerman, 2016). These responses reflect a dynamic, context-sensitive adaptation to anticipated threat, rather than delayed reaction to past trauma. Individuals exposed to continuous threat often fluctuate between internalizing symptoms such as fear, anxiety, somatization, and withdrawal, on the one hand, and externalizing behaviors including aggression, impulsivity, or hyperarousal, on the other, depending on their appraisal of safety and threat (Docherty et al., 2023; Hecker et al., 2017; Somer and Ataria, 2015). In such contexts, psychological functioning is shaped by the ongoing interplay of past experiences, current realities, and anticipated dangers, creating a circular loop of vigilance and adaptation (Eagle and Kaminer, 2013; Hecker et al., 2017).

To deepen this theoretical foundation, the study also briefly draws on Kira’s (2001, 2019, 2022) development-based trauma framework (DBTF) as a sensitizing concept, which highlights how trauma is transmitted intergenerationally and shaped by collective identity. DBTF defines trauma as threats not only to physical safety but also to one’s cultural belonging, personal agency, and sense of social integration. It recognizes that structural oppression, ethnic discrimination, and cultural marginalization are cumulative stressors that undermine emotional and developmental stability across the lifespan (Kira, 2019; Kira et al., 2013).

An important extension of DBTF involves the identity and existence salience in stress responses. When salient identities are threatened, appraisal and coping become intensified, increasing vulnerability to distress (Kira et al., 2011; Marcussen et al., 2004). Children’s appraisal and coping mechanisms are intensified when their ethnonational identity is criminalized or denied recognition, as is often the case under occupation. Individuals may also shift between personal and collective identities as an adaptive strategy, although this shifting can create new vulnerabilities (Kira et al., 2011). Over time, these experiences contribute to identity erosion, emotional dysregulation, and symptoms such as collective annihilation anxiety and diminished self-worth (Kira et al., 2012).

Empirical studies grounded in DBTF demonstrate that identity-based discrimination, military violence, and structural poverty are closely associated with increased symptoms of depression, PTSD, and somatization (e.g., Kira et al., 2013). These cumulative exposures result not only in psychological distress but also in profound violations of children’s developmental rights and autonomy. By situating children’s perceptions of insecurity within frameworks that view trauma as continuous, identity-driven, and socially embedded, this article contributes a context-sensitive lens for understanding youth experiences under occupation.

While CTS (Eagle and Kaminer, 2013) focuses on real-time adaptations to ongoing threat, DBTF (Kira, 2001, 2019, 2022) underscores the cumulative, identity-disruptive nature of trauma across the lifespan. Together, they provide a powerful framework for understanding the lived experiences of children under structural occupation. In this study, CTS serves as the organizing interpretive framework adopted after initial inductive coding; DBTF (Kira, 2022) is used *briefly* to clarify identity/existence-salient appraisals of threat (i.e., how threats to a salient ethnonational identity intensify stress responses). This dual framework is especially relevant in the Palestinian context, where fear and threat are rooted not only in individual exposure but also in ethnopolitical identity and a shared history of structural marginalization. Combined, CTS and DBTF emphasize the need to recognize ongoing stress as a critical determinant of mental health and adaptive functioning, while challenging traditional views that pathologize passive or withdrawn coping strategies.

### Psychological consequences of political violence for children in conflict zones

Palestinian youth’s experiences in East Jerusalem resonate with those of children in other conflict-affected contexts (Barber et al., 2016). Children living in conflict zones are frequently exposed to continuous sociopolitical violence. A growing body of research underscores how sustained and cumulative exposure to threat disrupts their emotional regulation, cognitive processing, and psychosocial development. Extensive evidence shows that perceived discrimination and structural violence consistently predict poor mental and physical health outcomes across multiple marginalized groups, including refugees, racial minorities, and children in militarized zones (Pascoe and Smart Richman, 2009; Williams and Mohammed, 2009).

A systematic meta-analysis by Agbaria et al. (2021) found that nearly 50% of Palestinian children and adolescents exposed to political violence in the West Bank and Gaza met diagnostic criteria for PTSD. From a comparative international perspective, Scharpf et al. (2023) conducted a network analysis of PTSD symptoms among 2,007 war-affected youth aged 6–18 in African countries, Iraq and Palestine, finding that across this diverse sample, hypervigilance, intrusive thoughts, and negative mood consistently emerged as central and highly interconnected symptoms. Subgroup analyses showed that in both children (6–12) and adolescents (13–18), these symptoms were not isolated but formed dense networks that reinforced each other, suggesting a cyclical and persistent structure of traumatic stress. In the Palestinian subsample, youth reported hyper-arousal and emotional dysregulation even in the absence of discrete traumatic events.

Scholars have also documented the normalization of abnormal conditions as a paradoxical coping strategy. For many Palestinian children, adapting to pervasive instability and militarized threat involves internalizing them as a new normal—reflecting both emotional desensitization and an attempt to preserve psychological continuity in contexts where safety is elusive (Nguyen-Gillham et al., 2008; Veronese et al., 2022).

Across several contexts, Palestinian youth exposed to violence reported elevated levels of psychological symptoms, reduced social competence, and maladaptive coping (Dubow et al., 2009). As mentioned, these psychological outcomes are further compounded by emotional desensitization. Children exposed to repeated violence may exhibit reduced emotional reactivity, which, while protective in the short term, is associated with increased externalizing behaviors and diminished empathy. Conversely, children who maintain emotional sensitivity are more prone to trauma-related symptoms, including intrusive memories, avoidance, and hypervigilance (Agbaria et al., 2021; Scharpf et al., 2023).

This dynamic interplay between emotional blunting and dysregulation reflects a disruption of normative emotional development. In line with this, Kumar et al. (2017) describe a “triple burden of violence” faced by children in conflict zones—exposure to armed violence, domestic abuse, and insecurity within displaced communities. In contexts like Palestine, children face not only direct harm but also a loss of community support and limited access to essential services, leading to chronic psychological distress and the normalization of violence (Veronese et al., 2022). Frameworks such as CTS and DBTF are therefore essential for capturing how chronic and identity-based threats erode children’s sense of safety, autonomy, and dignity (Eagle and Kaminer, 2013; Kira, 2001).

### The current study

East Jerusalem was unilaterally annexed by Israel in 1967, tripling the city’s municipal boundaries to incorporate land from 28 surrounding Palestinian villages. This annexation, deemed illegal under international law (Avni et al., 2022), resulted in the reclassification of Palestinian residents as permanent residents rather than citizens—a fragile status contingent upon proving one’s “center of life” remains within the city. Since 1967, over 14,200 Palestinians have had their residency revoked (Ir Amim, 2015), deepening their vulnerability to displacement and statelessness (Jefferis, 2012).

The 2002 construction of the Separation Wall entrenched East Jerusalem’s political and physical isolation, severing it from the rest of the West Bank and severely restricting movement, family unification, and access to essential services (B’Tselem and HaMoked, 2004). These restrictions constitute structural violence, deeply entangled in residents’ everyday experiences of racialized policing, home demolitions, unequal education access, legal precarity, and surveillance (Aly et al., 2025). Recent critical scholarship conceptualizes these processes as forms of “unchilding,” where Palestinian children are systematically denied rights, protections, and dignities afforded to children elsewhere (Shalhoub-Kevorkian, 2019; Tanous et al., 2022).

### Participatory approach

Building on the call for more qualitative, child-informed inquiry in politically constrained settings (Peleg, 2013; Veronese et al., 2022), the present study addresses the gap in research on how children and youth in occupied East Jerusalem experience and respond to continuous political and structural violence by examining their perspectives and giving them a voice. This study is part of a broader research project that also examined coping, psychosocial responses, and resilience among this population. It adopts a youth-centered and participatory approach, using a child and youth advisory group (CYAG) which consisted of seven adolescents; two boys and five girls, aged 12–19, recruited from different neighborhoods of East Jerusalem. The CYAG was trained to position as children co-researchers who actively shaped the inquiry (see Zedan and Gal, 2025). This approach is based on Lundy’s model on rights-based participatory principles that uphold young people’s agency and epistemic authority in knowledge production, and improve the applicability of findings for practice (Lundy et al., 2011). It was adopted because it aligns with children’s right to be heard and with the Lundy model’s emphasis on *space, voice, audience, and influence,* which is based on UNCRC, thereby reducing adult-centric bias and strengthening ecological validity (Lundy et al., 2011). Methodologically, partnering with youth improves credibility and practical relevance—youth help ensure culturally safe prompts, sharpen interpretation, and support trustworthiness (credibility/transferability) of qualitative findings (Lundy et al., 2011).

In this study, the CYAG served as advisory co-researchers and conducted interviews with professionals whose perspectives were used for triangulation. They received training, co-refined interview prompts, advised on ethics and recruitment messaging, and joined member-checking/interpretation workshops. They did not conduct interviews with their peers, access raw identifiable data, or handle recordings. Their involvement with transcripts was limited to selected, anonymized excerpts shared during interpretation workshops. To avoid overstating the design, we describe the study as participatory, rights-based, with a CYAG advisory partnership, rather than full participatory action research (PAR).

## Method and materials

### Participants

A community-based recruitment strategy was employed, combining online ads with in-person outreach at public libraries and youth centers. The author and the CYAG research team also collaborated with local youth organizations to facilitate participant recruitment. Adolescents who expressed interest in the study referred peers, utilizing a snowball sampling approach. For triangulation, two fathers and two mothers of participants, as well as four other professionals working with children and adolescents in East Jerusalem (a lawyer and three social workers), were recruited by the CYAG.

The final sample consisted of 24 Palestinian adolescents aged 12–19 years (*M* = 16.08, SD = 2.06), living in various neighborhoods of East Jerusalem. Most were female (*n* = 18, 75%). All were identified as Muslim and reported medium to high religiosity. They attended gender-segregated schools, some affiliated with the Israeli municipality of Jerusalem and others with the Palestinian Authority. The sample size was deemed adequate to achieve data saturation; we ceased recruitment when no substantively new codes were identified, consistent with qualitative research standards (Guest et al., 2006).

### Data collection

A semi-structured interview guide was developed, consisting of open-ended questions focusing on participants’ experiences with sociopolitical stressors and their coping strategies. These included interactions with police, military armed forces, settlers, and other Jewish individuals, as well as incidents of ethnic discrimination and racism. The guide was informed by existing literature and ongoing research and was piloted and refined in collaboration with the CYAG. It also included optional prompts to assist participants in recalling specific experiences and to elaborate on coping behaviors or psychosocial responses, though most of the incidents had naturally arisen. To maintain child safety and confidentiality, only the first author interviewed minors; CYAG members did not interview adolescents or access identifiable youth transcripts.

### Interview guide

The interview guide was collaboratively developed with CYAG to explore adolescents’ lived experiences of daily sociopolitical stress in East Jerusalem. It began with general questions about neighborhood conditions and everyday life, then moved to more focused prompts on interactions with authorities, experiences of discrimination, and perceptions of personal and collective identity. Particular emphasis was placed on understanding emotional reactions, meaning-making, and coping strategies. The guide was adapted throughout data collection, allowing for the refinement of questions based on emerging insights from earlier interviews. While the full guide addressed a range of themes, this article focuses specifically on participants’ perceptions of threat, insecurity, and their coping responses to sociopolitical stressors.

After providing informed assent and consent, the 24 participants were interviewed in Arabic by the first author between April and August 2024. The interviews lasted 45–105 min. Six interviews were conducted via Zoom according to the interviewees’ choice. The rest were conducted face-to-face in community youth centers and public libraries.

As mentioned, eight supplementary interviews were conducted with adults to enhance the credibility and contextual richness of the findings. The parental interviews were conducted by the first author via Zoom, while interviews with professionals were conducted face-to-face by trained CYAG members, under the author’s supervision; CYAG interviewers received focused training on ethics, addressing power balances, and interviewing skills. These interviews took place between August and December 2024 and lasted 30–45 min. While the primary analytical focus remained on the youth narratives, these adult perspectives contributed to method and source triangulation (Denzin, 1978), supporting the credibility of emergent themes.

### Data analysis and trustworthiness

All interviews were audio-recorded with consent, transcribed verbatim in Arabic, and, where relevant, translated into English with independent accuracy checks. Data were de-identified and stored on encrypted drives. Data analysis was iterative and inductive, following reflexive thematic analysis procedures. In a first cycle, the first author, using ATLAS.ti, conducted line-by-line coding of Arabic transcripts to preserve meaning, wrote reflexive memos, and maintained an audit trail; codes were developed inductively from the data (Braun and Clarke, 2006). In a second cycle, codes were collated into candidate themes using comparative reading across cases; after initial inductive coding, we adopted CTS theory as the primary interpretive lens to name and refine patterns, while checking that themes remained grounded in participants’ accounts (Braun and Clarke, 2019).

The triangulation interviews with parents and professionals were also coded thematically and used to support the credibility and depth of youth-derived themes. Codes across all sources were clustered into themes and subthemes, and findings were situated within the broader literature on youth navigating political violence. To ensure rigor and trustworthiness, we drew on Lincoln and Guba’s (1988) criteria of credibility, transferability, dependability, and confirmability. Reflexivity was practiced throughout, involving self-awareness, memo-writing, and group discussions to critically examine assumptions and positionalities. The lead researcher, a Palestinian citizen of Israel, conducted initial open coding and engaged in theme mapping and refinement to explore connections between categories. The analysis was refined collaboratively with the CYAG and supported by discussions with Palestinian and Jewish-Israeli researchers. Regular meetings and analytic memo-writing contributed to analytical depth and dependability. To support transferability, we provided thick descriptions of participants’ sociopolitical context and used rich quotations to illustrate findings. Confirmability was enhanced through transparent documentation of analytic decisions.

### Ethics statement

This study received ethical approval from the ethics committee of the author’s affiliated institution (protocol number: 2023HLE046). All CYAG activities were IRB-approved; identifiable data were not shared with CYAG. For minors, parental/guardian consent and youth assent were obtained; adult participants provided informed consent. Consent followed a clear explanation of the study’s purpose, significance, and the measures taken to ensure confidentiality and anonymity. Participants were informed that the study would not examine their political views or illegal actions. The limits of confidentiality (e.g., imminent risk of harm or other legally mandated reporting) were explained. They selected pseudonyms, and all identifying details were removed from the transcripts. They were also informed of their right to skip any question or withdraw from the interview at any time without consequence.

Prior to each interview, the researcher offered a verbal explanation of the ethical procedures, including how to navigate sensitive or potentially self-incriminating content. Participants were advised to frame such disclosures as experiences of others and to avoid using real names. If concerns arose during the interview, they were encouraged to pause the recording and consult with the researcher before continuing. To further support participant well-being, an optional concluding segment was offered in which the interviewer, drawing on their social work background, facilitated a brief, strengths-based conversation focused on coping strategies, sources of resilience, and positive relationships— with the aim of supporting participants and minimizing distress. At the end of each interview, participants were offered a short resource list for free-cost psychosocial support and community services.

## Results

Six interrelated themes emerged from the thematic analysis. All are subsumed under the umbrella theme of living insecurely under chronic traumatic stress.

### Perpetual threat in everyday spaces

Palestinian children and youth consistently describe their daily lives as imbued with violence and even death. This looming threat was not tied to specific actions but to their very identity as Palestinians, particularly those visibly marked by gender (e.g., being a young man or a girl in religious dress) or location. This ongoing threat is not only physical but also psychological, shaping their entire experience of public space. For instance, Shams, a 19-year-old girl from Al-Tur neighborhood, articulates how routine actions such as passing through military checkpoints are fraught with danger:

Insecurity is always present. We have no safety at all. Anyone can be shot at any time. […] when I pass through a checkpoint, I always think that something could happen. Any young person, […] or women wearing hijabs, could be accused.

This experience is also reflected in Ghonwa’s traumatic experience. This 19-year-old recounted how, when she was descending the stairs at Damascus Gate in the Old City – a busy area frequently patrolled by Israeli police – three police officers stopped her at a corner. They asked why she failed to respond when they had called her, demanded to see her ID, and questioned her about her presence in the area. She explained that she was heading home. The situation escalated when a female police officer attempted to physically assault her. She resisted by holding the officer’s hand. She insisted that she had not done anything wrong and asserted her right not to be touched or assaulted. When they tried to search her, she refused, telling them she would pull the blouse up so that they could see she had nothing on her.

I said, either they will shoot me or kill me, or they’ll plant something on me and fabricate charges against me. I was sure of it […] I was scared too. I spent two days without sleeping, not going out of the house, still afraid.

Alongside fear, Ghonwa expressed a deep sense of injustice, emphasizing that the emotional consequences were not limited to the immediate moment but accumulated over time with lasting psychological effects.

Sana, a 14-year-old girl from Kufr ‘Aqab, further illustrates how fear permeates even the most mundane activities – fear not only for herself, but also for others:

The first time I took the train alone, I was afraid something would happen, that they’d arrest everyone and put us in jail. I was young at the time. I was scared because there were soldiers carrying guns, and I was afraid they would do something. It wasn’t necessarily about me; there were also a few Arabs, and I was afraid they would do something to them.

Similarly, Samara (16, Azariya) shared how fear accompanied her across multiple public spaces on her routine route from beyond the military barrier:

[…] when I take the train, there are many Jews and police. My experience on the train is, of course, full of fear. Settlers might harass or beat me, and the police might stop me on the street, not just at the checkpoint. […] This fear of being searched or arrested is always present.

Parental accounts further underscored the theme of perpetual insecurity, particularly regarding children’s restricted mobility and exposure to sudden violence by Israeli forces in a Palestinian neighborhood. The mother of Hala’s (14, Al-Tur) reflected: “There’s no safety in the neighborhood. Even taking the kids to the neighborhood park after sunset can turn into clashes. I’d rather face racism elsewhere [in Jewish-Israeli areas of Jerusalem] than risk life-threatening violence nearby.”

### Adaptive hypervigilance

Children and adolescents described a persistent state of bodily and emotional alertness, shaped by the sense that danger could emerge anywhere—at home, in school, on the street, at checkpoints, or on public transportation. This omnipresent threat compelled youth to monitor and regulate their appearance, posture, and behavior as a survival strategy to avoid confrontation or suspicion. They adopt embodied coping mechanisms such as keeping their hands visible, lowering their gaze, altering their clothing, or holding objects like phones in certain ways. These are not only behavioral adaptations but expressions of internalized hypervigilance—a deep awareness that their very bodies and movements may be perceived as threatening by Israeli security forces. For example, Abu Gaith (16, Shuafat) described his routine efforts to avoid targeting:

A little bit of safety, I mean, on the train, and it already happened to me, especially if I’m wearing black or holding a bag, I will end up with three security men around me who will harass me… If I carry a bag, I keep it away from my hands. I do not put my hands in my pockets whenever I get on the train, and I stay holding my phone as a precaution so they do not come and harass me.

Yasmin (15, F, Al-Ram) described another common experience, that of a bystander. She witnessed some youth being beaten, and immediately imagined herself as the target, showing alertness and anticipatory fear: “I kept imagining the scene. I thought they, the Jews, were coming toward me. I was scared and kept talking to myself. I acted normal.”

Similarly, Omar (16, M, Beit Hanina) reflected: “I feel like one day something will happen to us, like if someone gets shot or there’s a problem. It could happen one day to someone I know, like one of my cousins.” Omar’s words reveal a pervasive sense of anticipatory fear and existential uncertainty, emblematic of growing up under continuous threat.

More broadly, Talin (14, Shuafat refugee camp) vividly described her life in Jerusalem as a space of captivity and threat requiring constant alertness:

We consider our life in these areas almost like a prison, with unleashed dogs waiting to attack. You have to be extremely alert and always carry something to protect yourself, like pepper spray… That’s the minimum. We thank God they still allow us even that.

Finally, Sara (18, Beit-Hanina) illustrated how threat is internalized to the point that being in a public space where a Jewish individual is present triggers involuntary recurring mental simulations of being targeted, falsely accused, or shot:

It is really frightening […] people get killed and then they are blamed… They’re shot by mistake but they [the authorities] do not admit it. They start saying things like, he was planning, maybe an attack. I thought, what if I end up in that situation […]? That whole scenario played out in my head within seconds. I found it kind of funny and illogical. But this scenario runs through my head a lot, especially when I’m in places where there are Jews.

While Sara acknowledges the irrationality of her thoughts, her reaction reflects mental rehearsal of catastrophic outcomes, a hallmark of CTS (Eagle and Kaminer, 2013). In environments shaped by structural violence and impunity, the line between possible and probable danger is blurred, and such rehearsals become part of an adaptive readiness.

This theme was further supported through triangulation interviews with parents. Samara’s mother described how the persistent uncertainty under occupation—particularly during the ongoing war—cultivates heightened vigilance: “We’re always under psychological pressure, not knowing what might happen next… you cannot predict how the policeman who detains you would act.” Her hyper-awareness extends to her children: “If they are late by half an hour, I keep calling… and if they do not answer, my mind spirals.”

### Collective and intergenerational transmission of stress

Beyond personal experiences, many youths internalized fear through stories, warnings, and collective memory. These secondhand exposures shaped their sense of danger, fueling avoidance behaviors and a persistent fear. As Abed (16, M, Beit Hanina) related:

My classmate, just two weeks ago, was sitting with his leg out; his house is next to the [Separation] Wall. Suddenly, the police raided and took him to Room 4, and beat him at midnight. He called me and said, “Come over, I’m exhausted; bring me food and water.” I went there and found his clothes torn, his face swollen; he could not walk, it was a mess. They put him there for eight hours. Room 4 in Neve Yaakov [police station], once you are taken there, you know you’ll be beaten, possibly killed. As soon as you enter, you hear people screaming. This is a nightmare for us in Beit Hanina.

Another witness, Jana (12, Ras al-Amud), recalled how, on her way to Al-Aqsa Mosque with her family, she witnessed a woman being beaten by Israeli forces, and wondered, “What if we were there? What if they had beaten me instead? There is no safety.”

Such accounts illustrate how vicarious exposure permeates the consciousness of the entire community, fostering a sense of shared identity-based vulnerability. This form of collective trauma amplifies the fear that any one of them could be the next victim, as articulated summarized by Samara:

[…] there’s no safety for anyone—whether it’s a child, a woman, a man, or a youngster. Everyone is exposed […] as long as you are an Arab and a Muslim as well. […] I see and hear about situations that happen, and on social media.

In terms of long-term consequences, Talin described the enduring impact of collective trauma on her behavior and worldview, and recalled past events of children at her age, such as the burning alive of 16-year-old Muhammed Abu Khdeir in 2014 (Ihmoud, 2015) and the imprisonment of 13-year-old boy Ahmad Manasra in 2015 (see Otman and Shalhoub-Kevorkian, 2023):

I was young at the time and did not fully understand, but as I grew older, I realized more—especially after Ahmad Manasra’s case. My parents also told me about Abu Khdeir… He was just sitting outside the mosque, and the settlers kidnapped him, tortured him, and burned him. What was his fault? He had not hurt anyone… It made me very scared. I started fearing for my life. If they could do that to a boy just sitting near his home, how could I, as a girl, dare go near their areas alone?

Another critical dimension of this theme is the intergenerational transmission of traumatic events, where parents’ anxieties shape the emotional environment in which children grow up. Al-Damir (19, Kufur A’qab) explains how fear becomes embedded through upbringing:

It becomes a sense of insecurity, which generates a sense of insecurity in others. For example, I did not feel unhappy or insecure, and my feelings were normal, but because of my parents and their general fear for us […], this fear is passed on until we grow up.

Abed explains this fear, “Our parents are very afraid for their children. They are always worried that their children would do something related to security or be accused of something. When someone is martyred […] they destroy the *family’s* house.”

Parental fears often translate into overprotection, and fear for all family members toward each other. Layla (14, F, Ras al-Amud) shared: “My mother is afraid to let my brother go out because he’s the only boy in the family. And we are also afraid for him.my parents are afraid for all of us.”

This theme also emerged in interviews with parents. Layla’s father described how the chronic stressors he experienced spilled over into his relationship with his daughters:

A father should be a role model. But when I’m stressed out because of the situation, I try not to take it out on my daughters. Even if I try not to take it out on my children, the pressure affects all of us […] We end up releasing our stress on each other.

This narrative underscores how ongoing sociopolitical pressure undermines parents’ capacity for emotional containment and affects overall family well-being.

Jana’s father reflected on a deeply traumatic event from his own adolescence:

At 17 or 18, I was on my way to help my brother at his office in Jerusalem. Soldiers stopped me and asked for my ID. When I told them I was a Palestinian-Arab Muslim, one of them mocked me, searched me, and falsely claimed I had a utility knife—he took it out of his own pocket. Five or six soldiers beat me badly, just because I said who I was. I did not go to work that day; my body was crushed. I still tell the story to my children, and they remember it. Even my little daughter gets scared and imitates the gestures. I cannot forget it, even after 30 years. This trauma has stayed with me, and now my kids carry it too. I try to make them understand that we have to endure this situation—it’s a policy meant to pressure us so we, in turn, pressure our children, and life becomes miserable. The kids understand this; they need to be aware that this is the reality we live in, and there’s no other option unless we leave completely.

These testimonies reflect how parental protection—meant to ensure safety—instills in youth a persistent sense of danger. Over time, this caution becomes part of the child’s emotional landscape, reinforcing hypervigilance, spatial avoidance, and a feeling that harm is always possible. These responses give way to deeper emotional adaptations, including suppression and withdrawal, as explored in the next theme.

### Emotional suppression and helplessness

Across interviews, the participants described emotional responses shaped by their chronic exposure to violence. A recurring thread was a state of emotional suppression and resignation, shaped by a persistent sense of powerlessness and the need to endure injustice in silence. Thus, rather than expressing acute fear, many participants conveyed emotional exhaustion, behavioral withdrawal, and detachment. Speaking out or reacting emotionally in public was seen as dangerous. As Aziza (17, F, Beit Hanina) shared:

I keep silent […] I pretend I do not see and ignore it every single moment. But inside, I’m truly upset, thinking: Why are you doing this? But I just stay quiet because there’s nothing I can do about it… I feel suppressed — why cannot I speak up? Why are you looking at me like that? Or why are you talking to me this way?

This suppression is a form of protective silence in an environment where expression could escalate risk. Several youth described how this self-silencing is accompanied by the normalization of abnormal conditions. Sara, for example, reflected:

There’s nothing we can change […] You just have to adapt. You try to live a normal life, which is not actually normal. We’ve come to see the checkpoint as normal: whether it’s crowded or not, open or closed — it’s like it’s normal. But it’s not.

Indeed, many interviewees noted that speaking up, reacting, or even exhibiting emotion in public spaces could provoke punishment or escalation. This was echoed by Abed, whose tone revealed both emotional fatigue and fatalism:

It’s not that I’m tired… I’m just fed up. Every time, they want to take us or beat us. That’s enough… My dad got used to it because I always cause him problems. He started saying, “Let them take you.” […] It became like a joke, a routine — we no longer see it as a big deal… It’s the occupation, and we go along with it. We cannot protest — we have seen what happens to those who resist. They get taken, imprisoned, tortured, and maybe even killed […].

Abed’s narrative reflects desensitization to state violence, as well as vicarious fear and shared trauma. This emotional resignation was often described alongside feelings of suffocation, anger, and despair, yet participants emphasized their need to cope silently. Tala (17, Beit-Hanina) spoke explicitly of the emotional toll of this resignation:

Living in Jerusalem, you constantly suppress your emotions. You cannot express what you feel — not even your opinion — because you are surrounded by people, and anything you say could put you at risk. So you stop talking about the situation; there’s no use.

When asked what this emotional silencing does to her, she responded: “It builds a lot of pressure… Seeing them [Israeli forces] every day fills me with anger, and then I feel helpless because I cannot do anything for my homeland.”

In addition to emotional suppression, youth described avoidance behaviors—withdrawing from social media, avoiding unsafe areas, or refraining from speaking to friends or family to protect others or themselves: “I try to hold it in. If I speak up or post online, I’ll end up harming my family, my father, and myself. So I avoid it, to stay out of trouble… I also do not dare go into their [Jewish] areas” (Talin).

In interviews with parents, emotional suppression also emerged as a necessary strategy for navigating repeated encounters with systemic control. Hala’s mother described the inner conflict of wanting to resist humiliating searches but choosing compliance:

There are times when you feel helpless… Refusing a search might lead to harsher treatment or longer procedures, as if saying no makes you guilty. So unfortunately, the only option is to comply, cut my losses, save time, and protect your energy—because it’s truly difficult.

Samara’s mother described her daughter’s emotional withdrawal: “She stays quiet or isolates herself in her room. She rarely talks—only when something extreme happens, like police entering the school or blocking the entrance.

These accounts reinforce youth and parents’ narratives of muted emotions and perceived futility in resisting. They illustrate how suppression is modeled and reinforced within the family as an adaptive survival response to chronic threat.

While this theme focuses on emotional suppression and powerlessness, it is important to note that some youth also described active and resilient strategies, including religious-spiritual grounding, cognitive reframing, social support, agency, and solidarity. These are explored in a separate article (Author, in preparation).

### Normalization of abnormality

Over time, the abnormal life under occupation became emotionally internalized, leading to a shift in how safety and danger are perceived. This theme explores how youth psychologically adapt to persistent insecurity, as danger becomes routinized and the distinction between fear and safety gradually dissolves. Several participants described how the ongoing threat in East Jerusalem ceased to evoke intense emotional responses, pointing to a broader process of normalization of abnormality. Shams, for example, openly rejected the idea of living in fear, even while recognizing that no place felt truly safe:

There’s no place where I feel safe… I’ve reached the point where it just does not matter anymore. Do whatever you want—I want to live my life. I will not lock myself in just because of this fear. It does not work for me anymore… We’ve seen so much war and killing since we were kids, it just does not affect us the same way.

Similarly, Majd (18, Old City) reflects on the frequent arrests and harassment of youth in her neighborhood, describing how fear and tension initially accompany such encounters but gradually give way to resignation: “When we go out at first, we feel tense, and then it becomes normal. At the beginning, there’s tension because it’s something new to you, and then that’s it—it just becomes the way it is for us.”

These reflections illustrate a form of emotional desensitization shaped not by healing, but by prolonged exposure. Fear does not disappear—it becomes muted, folded into daily life. Youth do not stop recognizing danger; rather, they recalibrate their emotional responses and redefine emotional normalcy in order to reclaim their autonomy and continue navigating unsafe environments: “I do not feel fear anymore when I get stopped [by police] or when something frightening happens. I’ve gotten used to it. As far as fear goes, I’ve been used to it for a long time—it just becomes normal” (Abu Gaith).

Saja, a social worker employed in a boys’ school in East Jerusalem, described how repeated exposure to state violence becomes embedded in children’s everyday reality and identity development:

Children as young as 4–5 witness the arrest of their parents, see soldiers raiding their homes, and come to understand their identity in these moments. They know they are on their land, but they do not feel the safety or belonging they are supposed to feel.

The social worker’s account adds depth to the theme by showing how early and deeply such experiences affect children’s sense of security and identity—underscoring how spaces of care, such as the home, are destabilized by chronic threat. This normalization process blurs the line between safety and danger, shaping children’s worldview and sense of self from a very young age.

Mazen, a lawyer who represents families in cases related to residency and citizenship, reflects:

The checkpoints slow us down, but that’s not enough of a reason to say they are holding us back. My appointment’s at seven—I leave at six. That’s just how life is. We got used to it. […] It got to a point where if a soldier stops a boy, the boy starts searching the soldier. […] Without the humiliation they put us through, we would not know how to live—we’d turn on each other.

Here, the violence is trivialized as “no excuse” for being late and simultaneously acknowledged as so pervasive that individuals learn to accommodate it, even to the extent that youth no longer show fear and respond with defiance. Structural violence is thus internalized, inscribed into daily behavior and collective consciousness.

### Distrust in protective systems

A final theme that emerged across interviews was the profound erosion of trust in institutions and social anchors conventionally expected to provide protection or support. For Palestinian youth in East Jerusalem, these systems—ranging from legal institutions and police to schools, NGOs, and even peers—were not only ineffective but frequently perceived as complicit in oppression or simply powerless.

Many participants described a sense of futility and even risk in seeking justice in harassment cases. Legal systems were viewed as biased, performative, or outright harmful. Nadine (18, F, Shuafat) explained:

If you react or respond [to settlers’ harassments], it turns against you. I just keep walking to avoid unnecessary trouble, because if the police come, you’ll be the one prosecuted and punished. Even legally, we cannot do anything. If we file a complaint, they’ll just drain your energy, and in the end, nothing happens.

Similar disillusionment was shared by Sadin (19, Old City), who recounted how police closed her friend’s sexual harassment case within hours, although she had identified the perpetrator: “They told her, ‘What matters is that you are okay’, and closed the case.” This impunity extended to encounters with soldiers and settlers. As Omar put it, “If a soldier does something, nothing happens to him. But if we defend ourselves, we are the ones to blame.”

Moreover, the risks involved in expressing Palestinian identity or political views further deepened mistrust. For many youth, national pride became a source of fear:

Expressing our Palestinian identity has become a threat. We’ve started to express less, fear speaking, and distrust those around us… Even a social media post praying for Gaza can be labeled as incitement. You could lose your job, your rights, or be arrested. It’s terrifying (Sadin).

Sadin’s fears were both real and shared by others (Molana-Allen and Cebrián Aranda, 2023). Other youth described concrete risks of entrapment:

You have to be careful with every word you say and who’s listening, […] my friend was arrested and beaten so badly, he turned purple. He was just talking near someone dressed like a local—turned out to be an undercover agent [*Mista‘rib*]. There’s no safety here (Abed).

Beyond state structures, trust within the community itself was eroded. Several youth described fear of surveillance and betrayal from within their social circles: “Even with friends, you stay silent—afraid their family might be informers. We’re not just silencing ourselves because of Israelis. We’re losing trust among each other” (Tala). Thus, informal social support, typically a critical buffer against chronic stress, is compromised: “You’re always anxious, even with your closest friends. What if they are working with the Israelis? You hide your identity. You silence yourself—even among your own people” (Tala).

This climate of fear extends into institutional settings. Nadine explained why, when she feels distressed, she avoids seeking psychosocial support from NGOs or mental health professionals:

I’d feel restricted in what I can say. It’s better to talk to my family and friends freely. These organizations might harm more than help. We cannot express ourselves, not even on social media — so how could I open up to outsiders?

Jessie (18, F, Beit Hanina), who experienced distress during the war on Gaza, echoed this sentiment: “They wanted me to talk to the school counselor, but I knew what she’d say— ‘we cannot do anything; everything is monitored’. Teachers are cautious. They fear arrest over a single wrong word.”

This multilayered collapse of trust created a pervasive sense of vulnerability and emotional isolation. Community organizations, school-based support, and peer networks no longer offered refuge.

## Discussion

This study explored how children and youth in East Jerusalem perceive and respond to sociopolitical traumatic stress under conditions of prolonged occupation, and specifically during the ongoing war on Gaza. By applying a participatory, rights-based approach to qualitative inquiry, this study deepens our understanding of how continuous, identity-targeted stressors are internalized, embodied, and reproduced across generations. During the ongoing Gaza war, these dynamics intensified, affecting daily functioning and magnifying developmental costs.

The findings reveal that the children’s psychological and emotional worlds are shaped by continuous and institutionalized violence, which generates a perpetual sense of threat that transcends space, age, gender, and generation. They live in a state of constant alertness, shaped by experiences of ongoing and anticipated violence, where safety-oriented vigilance becomes a survival mechanism. Their coping responses reflect both direct personal exposure and vicarious collective and intergenerational trauma, rooted in a shared identity as Palestinians and a deep awareness of systemic oppression.

The participants reported profound feelings of helplessness, resignation, emotional suppression, and behavioral avoidance, illustrating how continuous exposure to trauma undermines their sense of control and disrupts normative development. The experience of growing up in an unsafe world—where the threat is chronic and spatially pervasive—led many to cognitively normalize abnormal life conditions as a means of survival, adaptation, and emotional endurance.

Finally, the participants expressed a profound loss of trust in institutions meant to protect them. These forced adjustments echo the theory of CTS (Eagle and Kaminer, 2013), where threat is ongoing and reinforced by structural violence, leaving children without closure or protection.

### Thematic cycle of continuous traumatic stress

This study yielded six interconnected themes, each reflecting and extending the core components of CTS theory (Eagle and Kaminer, 2013). Rather than isolated experiences, the findings point to a chronic, anticipatory, and socially embedded form of trauma that is shaped by both structural and emotional landscapes.

The themes unpack how continuous political violence is experienced across physical, emotional, temporal, and relational domains. The first two address the spatial and embodied dimensions of threat. The third explores the collective and intergenerational transmission of stress through memory and shared suffering. The fourth and fifth examine internal consequences of this environment: emotional suppression and the normalization of violence that blurs boundaries between safety and threat. The final theme depicts distrust in protective institutions and community anchors. Together, these themes reveal how structural violence becomes cyclical and developmentally disruptive, necessitating trauma frameworks that account for sustained, cumulative exposure. Below, we map our findings onto the four core hallmarks of CTS.

#### Ongoing, real, and anticipated threat

The first hallmark of CTS is the persistent exposure to real and anticipated danger, often without a foreseeable end or recovery period (Eagle and Kaminer, 2013). This chronic threat undermines any sense of safety or predictability in everyday life. In our study, this was most clearly reflected in Theme 1: Perpetual Threat in Everyday Spaces, where youth described pervasive feelings of vulnerability and fear across routine settings, and even within their homes. While Theme 1 reflects spatial and environmental precarity, Theme 4: Emotional Suppression and Helplessness reveals how this chronic exposure manifests in youths’ internal world — through emotional numbing, helplessness, and restricted self-expression out of fear and as adaptive survival strategies. Both themes reflect the emotional and physical insecurity in everyday spaces. This interplay between external danger and internal adaptation illustrates how continuous threat extends beyond behavior into emotional regulation, gradually eroding resilience. These results align with research on youth in conflict zones, where chronic exposure to political violence leads to heightened emotional dysregulation, dissociation, and long-term psychological impairment (e.g., Agbaria et al., 2021; Sagi-Schwartz, 2008; Scharpf et al., 2023; Wentz et al., 2023).

#### Difficulty differentiating real, potential, and imagined threats

A second core hallmark of CTS is the blurring of boundaries between immediate, potential, and imagined threats, which disrupts an individual’s ability to recover, anticipate safety, or distinguish between danger and normalcy (Eagle and Kaminer, 2013). In such contexts, safety-oriented vigilance becomes a baseline state of being. This often results in anticipatory anxiety, emotional numbing, and avoidance, not as signs of pathology, but, again, as adaptive survival strategies (Hecker et al., 2017). This process was captured in Theme 3: Collective and Intergenerational Transmission of Stress. Our participants frequently referenced inherited fears and anticipatory dread, often conveyed through family stories, behaviors, and emotional tones. They also reported intense fears for both their own and their loved ones’ safety. This resonates with Kira’s (2001, 2019, 2022) development-based trauma framework (DBTF), which conceptualizes trauma as cumulative and intergenerational—especially when linked to a group’s targeted identity. The trauma is not bound to a single event, but becomes embedded in the family’s emotional legacy and collective political context.

This temporal dislocation—where trauma from the past and fear of future violence are continuously present – makes it difficult to differentiate real, potential, and imagined threats, forcing the state of alertness and vigilance depicted in Theme 2. where participants described being in a constant state of bodily readiness—exhibiting heightened startle responses, vigilance to environmental cues, and anticipatory scanning for signs of military or police presence. This challenges conventional trauma models that frame such responses as maladaptive, and instead positions them as embodied forms of self-preservation. These findings align with broader evidence demonstrating that cumulative exposure to political violence leads to increased psychological distress among Palestinian youth. Haj-Yahia et al. (2021), for example, found that the more Palestinian adolescents were exposed to political violence, the more they exhibited posttraumatic stress symptoms across the three core dimensions of intrusion, avoidance, and arousal (see also Kira et al., 2013; Huesmann et al., 2022).

#### Absence of external protective systems

A third core component of CTS is the lack of access to reliable protective systems. When institutions meant to provide safety—such as the police, legal systems, schools, or social services—are either ineffective, absent, or complicit in harm, individuals experience compounded vulnerability (Eagle and Kaminer, 2013). This was strongly reflected in Theme 6. Youth repeatedly expressed mistrust and disillusionment with institutions meant to provide support or protection. These systems were not merely ineffective, but (perceived as) complicit, punitive, or surveillant. This institutional breakdown exacerbated their sense of helplessness and isolation. Rather than buffering stress, these systems became sources of retraumatization. This aligns with Shalhoub-Kevorkian’s book (2019), describing how Palestinian youth experience legal and educational systems as mechanisms of control, rather than safety. It also resonates with Kira’s (2001, 2019) notion of identity trauma, wherein the repeated denial of institutional protection is experienced as an assault on one’s existential and collective identity.

The absence of trustful protective systems feeds back into Theme 4, as youth learn to detach emotionally to manage persistent fear or stay silent to avoid trouble. They grow up knowing that their body, presence, or even voice may be viewed as a threat by powerful others. Such responses are understood as contextually rational and socially learned forms of coping (Eagle and Kaminer, 2013). However, these strategies also signal the erosion of developmental agency, reflecting learned helplessness, and illustrating the cyclical nature of CTS.

#### Changes in meaning, identity, and relationships

The fourth hallmark of CTS involves deep disruptions in meaning-making, identity, and interpersonal trust. Prolonged exposure to threat can shift youth’s perception of what is normal, blur moral and emotional boundaries, and corrode trust in social institutions and relationships (Eagle and Kaminer, 2013). Over time, such shifts can lead to emotional numbing, moral disengagement, and the normalization of abnormality, fundamentally altering how individuals relate to themselves, others, and their sociopolitical environment (Huesmann et al., 2016; Veronese et al., 2022). This transformation was captured in Theme 5: Normalization of Abnormality, by blurring of safety and threat, where youth described becoming desensitized to conditions of structural violence. This echoes Eagle and Kaminer’s (2013) notion that chronic exposure erodes the capacity for outrage, forcing children and adolescents to reinterpret violence as part of the everyday landscape. It requires young people to adapt by redefining what safety, threat, and agency mean in their lives. This shift in meaning parallels findings from studies on youth in protracted conflict zones, where ongoing exposure to normalized violence leads to emotional flattening, social mistrust, and even endorsement of aggression as a survival strategy (Docherty et al., 2023; Dubow et al., 2009; Huesmann et al., 2016, 2022).

These moral and psychological shifts are also compounded by the experiences described in Theme 6. Participants described losing trust not only in formal systems but also in their nearest social circles, often driven by fear that these individuals might be undercover agents [*Mista‘ribin*], informers, or collaborators. These experiences cultivated a pervasive sense of suspicion and vigilance, contributing to a persistent perception that anyone could be a threat. As found in an earlier study, layered insecurities undermine social cohesion, making everyday life a site of struggle (Hammoudeh et al., 2016). This reinforces the CTS hallmark of shifting meaning, identity, and interpersonal trust. It represents a fundamental disruption in how youth perceive relationships, safety, belonging, and the world around them.

Continuous exposure to betrayal, militarization, and deception reconfigures youth’s emotional and cognitive schemas (e.g., hostile-world views), often leading to anticipatory fear, anxious arousal (Huesmann et al., 2016, 2022), and a diminished sense of relational safety (Cummings et al., 2017). These are part of a collective matrix of unresolved and repeated trauma, where social ties themselves become sites of potential harm. This reflects what Eagle and Kaminer (2013) describe as the breakdown of relational trust—a critical component of CTS. It also aligns with Kira’s (2001, 2019) DBTF, in that the failure of systems to recognize or protect a targeted identity group becomes itself a source of trauma. The erosion of institutional trust described here is an adaptive realization that institutions are structurally misaligned with youth’s rights and needs. This adaptation—while emotionally costly—demonstrates an acute political consciousness and reflects a collective survival strategy in the face of systemic betrayal. As CTS theory posits, such disintegration of protective systems compounds the psychological burden and undermines youths’ capacity to seek safety, justice, or collective belonging (Eagle and Kaminer, 2013). Ultimately, these findings reveal that for Palestinian youth in East Jerusalem, the very systems meant to offer protection have become sources of additional harm. Silenced at school, surveilled in public, and mistrustful in private, young people face continuous threat and the collapse of communal safety nets. The result is a form of structural abandonment that exacerbates their distress and compounds their isolation.

*Overall,* each theme reinforces the others, forming a self-perpetuating spiral of survival under conditions of unrelenting insecurity. By anchoring these findings in CTS and DBTF, the analysis illuminates how Palestinian youth adapt to an ecosystem of threat that is spatially unbounded, identity-driven, and politically entrenched. Their experiences underscore the psychosocial consequences of structural violence along with its legal and ethical dimensions.

## Conclusion

The findings of this study reveal that the emotional exhaustion, hypervigilance, and normalization of danger described by Palestinian children and youth in occupied East Jerusalem reflect not only psychological distress, but also systematic violations of their rights under the CRC (United Nations, 1989) —including the rights to protection, development, dignity, and emotional well-being. These lived experiences, intensified during wartime, underscore how ongoing political violence erodes the foundational conditions for a safe and nurturing childhood. This study contributes to the expanding scholarship on structural trauma by empirically grounding the concept of unchilding (Shalhoub-Kevorkian, 2019) in the lived experiences of Palestinian youth in East Jerusalem. It illustrates how legal, political, and bureaucratic violence are internalized as chronic fear, emotional withdrawal, and anticipatory vigilance, stripping rights holders of legal and developmental recognition.

This study also offers a participatory, rights-based methodological approach to co-producing knowledge with youth in high-risk, politically repressive settings. Thus, both methodologically and theoretically, it reinforces the urgent need to reconceptualize children’s protection and development not as future-oriented aspirations, but as immediate rights, and to enable children’s agency and participation even within environments of profound structural injustice.

### Limitations and future directions

This study offers in-depth insights into the lived experiences of Palestinian youth in East Jerusalem under conditions of continuous traumatic stress. However, several methodological and contextual limitations should be acknowledged. First, recruiting participants was particularly challenging due to the ongoing war, heightened surveillance, and restrictions on freedom of expression. Excessive policing and fear of potential repercussions led some parents and youth, especially boys, to decline participation, particularly given the study’s sociopolitical focus. This may have affected the gender balance and scope of perspectives represented. Moreover, concerns about safety likely influenced what participants felt comfortable sharing. To protect both the youth and researcher, participants were encouraged to describe certain politically sensitive experiences as those of “others,” which may have introduced narrative distancing and enabled greater candor.

Second, the sociopolitical and legal context of East Jerusalem, marked by fragmented jurisdiction, surveillance, and structural inequality, is unique. As such, the findings may not be directly transferable to Palestinian youth living in other contexts, such as Gaza, the rest of the West Bank, sovereign Israel, or diaspora communities.

Third, while all interviews were conducted in Arabic and translated into English with attention to preserving original meanings, some culturally embedded expressions—particularly those reflecting trauma, resistance, or emotion—may have lost nuance in translation. However, efforts were made to retain participants’ voices in both quoting and analyzing their narratives.

Finally, this study reflects youth experiences during a specific period of heightened violence. It does not trace the long-term trajectories of coping or resilience, nor does it assess the mental health outcomes associated with different coping strategies. While participants shared coping mechanisms they employed, this qualitative design did not aim to evaluate their effectiveness or outcomes.

Future quantitative mixed-methods research could further explore how sociopolitical stress and coping experiences evolve and intersect with psychological wellbeing over time. Comparative studies with Palestinian youth in the West Bank and sovereign Israel are also needed to examine variations across political and legal contexts. Applying CTS-informed, rights-based frameworks to other conflict-affected populations, with attention to intergenerational trauma, resilience, and legal advocacy, can broaden the relevance of these findings. It is also recommended to amplify vulnerable children’s voices through participatory approaches that position them as co-researchers in the production of knowledge.

### Implications for policy and practice

Psychosocial interventions with children under occupation must go beyond symptom management to address root causes of ongoing traumatic stress, including militarization, discriminatory policies, and the denial of basic rights. Mental health responses should recognize emotional suppression and withdrawal (also) as collective adaptive strategies, rather than individual deficits. At the same time, interventions must strengthen children’s capacity to regulate distress while increasing predictability in their daily environments. This can involve helping schools and community programs maintain steady routines, offering low-stigma opportunities for children to connect with trusted adults for psychological support, and ensuring clear referral pathways so that urgent risks are addressed quickly and appropriately.

Clinically, survival behaviors should be validated as contextually adaptive, while practitioners introduce gentle regulation supports and safe micro-rituals to broaden coping repertoires. Where safety cannot be guaranteed, schools and services can create small anchors of stability—such as reliable check-ins, contingency planning, and rapid response pathways—to help children experience minimal consistency in otherwise unstable conditions. Protecting developmental growth also requires safeguarding time for play, peer connection, and exploration, recognizing these as essential for resilience rather than optional extras. At the family level, services should scaffold caregivers as emotional security anchors by equipping them with simple stress-regulation tools and ongoing support to reduce the spillover of fear and helplessness.

Integrating psychosocial support with rights-based advocacy is essential. Frontline clinicians, teachers, and school counselors should not only provide emotional support but also document systemic barriers children face—such as school disruptions, mobility restrictions, and militarized encounters—in de-identified ways. Community-based organizations and NGOs can then act as protection linkages, ensuring families are connected to legal aid, housing, or child-protection resources. International agencies, including UNICEF and Save the Children, play a critical role in amplifying these reports and pressing policymakers to ensure demilitarized spaces, equitable access to services, and enforcement of children’s rights under the CRC.

Collaboration is key. Psychosocial providers and advocacy groups should work together through joint case conferences and referral systems, so that children’s distress is addressed both clinically and structurally. Training clinicians and educators in child-rights frameworks—not only trauma-informed care—enables them to serve as caregivers, witnesses, and advocates simultaneously. Researchers can generate evidence that elevates children’s voices in policy debates, while youth advisory groups ensure that interventions remain grounded in lived realities.

As Peleg (2013) argues, safety must be redefined not only as survival but as the capacity for agency, decision-making, and developmental growth. Protecting children under continuous threat, therefore, requires reframing clinical work as both healing and defending rights. This means not only reducing exposure where feasible and ensuring predictable communication routines, but also creating conditions where children can live as children—spontaneous, playful, connected to peers, and future-oriented.

### Concluding reflection

Despite the persistent threat they face, the children and youth in this study demonstrate remarkable resilience, insight, and moral clarity. Their voices remind us that healing and justice are clinical and political goals, but also deeply human ones. In contexts of prolonged conflict, protecting children must mean more than minimizing harm — it must involve creating conditions in which they can grow, dream, and shape their futures with dignity. The responsibility rests with all of us—clinicians, educators, policymakers, researchers, international actors, and communities. Youth voices show that everyday protection (predictable routines, supportive relationships, safe spaces for play) and broader advocacy (challenging militarization, defending rights, amplifying voices) are inseparable. Honoring their resilience means moving beyond documenting harm to transforming the systems that perpetuate it. This collective duty demands courage, persistence, and solidarity so that the dignity and development promised in the CRC become a lived reality for children under occupation.

## Data Availability

Due to confidentiality and sensitivity of the topic, full transcripts are not publicly available. Additional anonymized materials may be provided by the author upon reasonable request.

## Footnotes

^1^We use “children and youth,” “children,” “youth,” “adolescents,” and “young people” interchangeably, to refer to boys and girls under the age of 18–19.
